# Bayesian spatial analysis of incomplete vaccination among children aged 12–23 months in Nigeria

**DOI:** 10.1038/s41598-024-57345-y

**Published:** 2024-08-07

**Authors:** Lanre Quadri Ahmed, Ayo S. Adebowale, Martin E. Palamuleni

**Affiliations:** 1 Department of Epidemiology and Medical Statistics, Faculty of Public Health, College of Medicine, University of Ibadan, Ibadan, Nigeria; 2 Population and Health Research Entity, North-West University, Mafikeng, South Africa

**Keywords:** Incomplete vaccination, Children, Vaccination coverage, Bayesian, Spatial, Nigeria, Health care, Risk factors

## Abstract

High childhood disease prevalence and under-five mortality rates have been consistently reported in Nigeria. Vaccination is a cost-effective preventive strategy against childhood diseases. Therefore, this study aimed to identify the determinants of Incomplete Vaccination (IV) among children aged 12–23 months in Nigeria. This cross-sectional design study utilized the 2018 Nigeria Demographic and Health Survey (NDHS) dataset. A two-stage cluster sampling technique was used to select women of reproductive age who have children (n = 5475) aged 12–23 months. The outcome variable was IV of children against childhood diseases. Data were analyzed using Integrated Nested Laplace Approximation and Bayesian binary regression models (α_0.05_). Visualization of incomplete vaccination was produced using the ArcGIS software. Children’s mean age was 15.1 ± 3.2 months and the median number of vaccines received was four. Northern regions contributed largely to the IV. The likelihood of IV was lower among women aged 25–34 years (aOR = 0.67, 95% CI = 0.54–0.82, p < 0.05) and 35–49 years (aOR = 0.59, 95%CI = 0.46–0.77, p < 0.05) compared to younger women in the age group 15–24 years. An increasing level of education reduces the risk of odds of IV. Other predictors of IV were delivery at the health facility (aOR = 0.64, 95% CI = 053–0.76, p < 0.05), and media exposure (aOR = 0.63, 95%CI = 0.54–0.79, p < 0.05). Mothers’ characteristics explained most of the variability in the IV, relatively to smaller overall contributions from the community and state-level factors (p < 0.05). The level of IV against childhood diseases was high in Nigeria. However, disparities exist across the regions and other socioeconomic segments of the population. More efforts are required to improve vaccination sensitization programs and campaigns in Nigeria.

## Introduction

Vaccination is used in the prevention of contagious diseases, particularly among under-five children who are vulnerable to many childhood diseases and infections. It is a critical strategy for child survival as well as achieving the third Sustainable Development Goal (SDG), which aims to reduce under-five mortality to fewer than 25 per 1000 births by the year 2030^1^. Worldwide, immunization programs averted more than 2 million deaths annually but many children are still missed^2^. World Health Organization reported that globally, more than 1 billion children have been vaccinated over the last decade and that the uptake of new and underused vaccines is increasing^2^. Despite this remarkable progress, nearly 20 million children each year have insufficient access to vaccines^2^. In some countries, progress has paused or even fallen, and there is a real risk that the past achievements made on this development may be compromised. In Africa, 1 in every 5 children is often unvaccinated against childhood diseases and an estimated 2.7 million mortality was recorded annually among children as a result of vaccine-preventable diseases and majority of these deaths occur in sub-Saharan Africa^2^.

However, over the past three decades, African immunization programs have made tremendous effort to ensure that the target threshold set by the WHO is accomplished^3^. Unfortunately, the coverage remains low for some recommended childhood vaccines^1^. In 2014, only Zimbabwe was found to have met the Global Vaccine Action Plan threshold of 80% or higher coverage of the DTP3 vaccine out of all the countries in sub-Saharan Africa^4^. In Nigeria, numerous immunization campaign programs have been implemented by the Federal Government in conjunction with some international agencies. Nigeria adopted the WHO Expanded Program on Immunization (EPI), to cover preventable childhood diseases such as tuberculosis, poliomyelitis, diphtheria, pertussis, tetanus, hepatitis B, yellow fever, and measles. The EPI emphasizes that a child receives 1 dose of Bacille Calmette Guerin (BCG), 4 doses of oral polio-containing vaccine (PCV), 3 doses of diphtheria, pertussis, and tetanus (DPT) vaccine, and 1 dose of measles-containing vaccine (MCV). Additionally, the Polio Eradication Programme, yellow fever, Measles, and Meningitis Campaign programs were held recently in some states in Nigeria and this has increased the immunization levels in recent times. Although, Nigeria has made some progress in terms of reduction in under-five mortality which could be attributed to an increase in childhood vaccine uptake, about 4.3 million children still miss out on vaccinations yearly^3^.

About 17.1 million children did not receive an initial dose of the DTP vaccine and an additional 5.6 million are partially vaccinated, and more than 60% of these children live in 10 countries including Nigeria^2^. The percentage of children aged 12–23 months who received all basic vaccinations increased from 25% in 2013 to 31% in 2018^5^. According to 2018 NDHS report, the prevalence of complete, incomplete and non-immunization of childhood vaccination in 2013 are respectively 25%, 54% and 21% while those reported in 2018 are respectively 31%, 49.9% and 19%^5^. These trends though indicate relative progress over the previous years. but are still far behind SDG3, for which the target is achieving more than 90% coverage of all basic vaccinations among children aged 12–23 months.

Previous studies^6–8^ on immunization issues are mostly based on logistic regression without accounting for geographical clustering. Based on the efforts national and international agencies have put into ensuring national coverage, it is pertinent to assess the incompleteness of childhood vaccines uptake, identify geographical locations lagging, and understand the effect of factors on vaccination patterns across the geographical location, to enhance economic productivity. Therefore, this study took into consideration, mothers who reside in states where exposure to unmeasured similar social environment and health services access occur, so it is possible that there might exists spatial correlations across different communities, states and geopolitical regions.

The objectives of this study are to: examine the spatial patterns of vaccination coverage among children 12–23 months of age and identify the individual, community, and state-level factors associated with incomplete vaccination. The diverse cultural groups in Nigeria have implication for access to health care including childhood vaccination. The 1996 trial in Kano where five of the children given trovafloxacin and six who were given ceftriaxone died remains a barrier to childhood immunization uptake in the Northern part of Nigeria. Understanding the spatial variation in vaccination coverage in the context of states or administrative units covered is important for evaluating the performance of our vaccination programs. Thus, Bayesian regression models were employed to quantify the spatial risk of childhood vaccination as well as associations between incomplete childhood vaccination and a range of factors, and other fixed effects on childhood vaccination.

**Figure 1:**
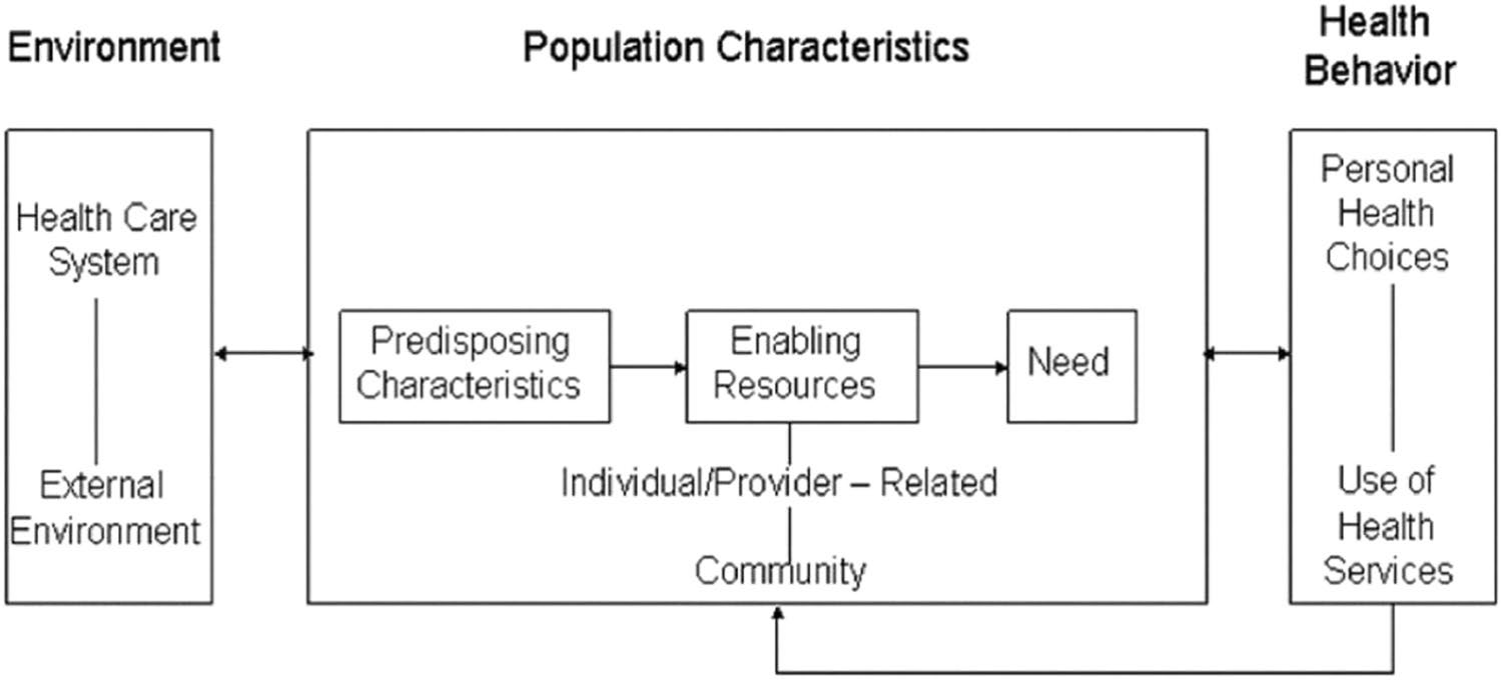
Conceptual framework adopted from Andersen’s health care behavioral model.

The conceptual framework for this study was based on Andersen's healthcare utilization and the Ecological Model as depicted in Fig. 1. Andersen Model was created to address disparities in healthcare access from the perspective of individual decisions made as a result of socioeconomic disparities^9^. The model posited that the usage of health services is determined by three dynamics: predisposing factors, enabling factors, and need. Predisposing factors can be characteristics such as race, age, and health beliefs. The enabling factors could be family support, access to health insurance, one's community, and so on. Need represents both perceived and actual need for health care services. This model has been used in the past for predicting healthcare utilization among population subgroups, and it takes into consideration of the minority groups, the rural dwellers, and the population group who may have challenges accessing healthcare. Similar to this model is the Social-Ecological Model which addresses health behaviors from the perspective that the observed health outcomes can be attributed to factors that may be analyzed at multiple levels which may go beyond individual characteristics. It considers the complex interplay between individual, relationship, community, and societal factors. This model suggests that interventions aimed at different levels may have a greater influence on health outcomes than interventions instituted at a single level^10^.

## Methods

### Study area

This study was conducted in Nigeria. The country has six geopolitical zones, which when subdivided at the secondary administrative level gives overall 36 states and the Federal Capital Territory (FCT). The states and FCT are further subdivided into 774 Local Government Areas (LGAs), and each LGA is divided into political wards. The country is made up of over 250 ethnic groups with a variety of cultures, languages, and traditions. The dominant ethnic group in the northern part of the country is the Hausa-Fulani, most of whom are Muslims. The Yoruba people are predominant in the southwest, half of whom practice Islam, and the other half are Christians. The Igbo are found in the southeastern region and are predominantly Christian^5^. There are also different minority ethnic groups across the regions. Detailed information about the study area can be found on https://dhsprogram.com/publications/publication-fr359-dhs-final-reports.cfm.

### Study design and population

The study was cross-sectional in design and based on the secondary data analysis (child recode) of the 2018 Nigeria Demographic and Health Survey. It was a nationally representative household sample survey for Nigeria. The 2018 NDHS included all women aged 15–49 years in the sample households. However, a section was devoted to the original questionnaire to address health-related issues about under-five children. This information was provided by the mothers of these children during the data collection. The current study focused on children aged 12–23 months.

### Sampling technique

A two-stage cluster sampling method was used for data collection. In the first stage, 1400 clusters were selected with probability proportional to cluster size. The survey was successfully carried out in 1389 clusters across the country, a household listing procedure was carried out in all the selected clusters, and the resulting lists of households were used as a sampling frame where households were randomly selected for the interview. In the second stage, 30 households were selected in every cluster using systematic sampling, resulting in a total sample size of 5475. Detailed information about the sampling and data collection methods used during the survey is available at https://dhsprogram.com/publications/publication-fr359-dhs-final-reports.cfm.

### Data source and extraction

The 2018 NDHS dataset, which is publicly available to all registered users, was requested from the Measure DHS (Demographic Health Survey) program website (https://www.dhsprogram.com). The available information about basic childhood vaccinations and their potential predictor variables were extracted. The geo-package of Nigeria's administrative areas at all levels of sub-division was used to identify hot spots of incomplete vaccination.

### Study variables

#### Outcome variable

The outcome variable was immunization status among children aged 12–23 months. According to the vaccination schedule in Nigeria, a child is considered fully immunized if he or she has received Bacille Calmette-Guerin (BCG) vaccination against tuberculosis and one dose of oral polio vaccine which are given at birth; three doses of DPT vaccine to prevent diphtheria, pertussis, and tetanus; another three doses of oral polio vaccine (OPV); and one dose of measles vaccine. These vaccines should be received during the first year of life^11^. Although, it is expected that within the first 12–23 months of life, all basic vaccinations should have been received by a child and this child is said to be completely vaccinated if he had received all of the immunizations recommended in the official immunization schedule^2^. To determine the immunization status, a new variable was generated for each of the vaccines and was regrouped as 1 for the uptake of a particular vaccine and 0 if a such vaccine was not received. Another variable was created to depict the total vaccine uptake for each child as follows:

$$ {\text{Immunization }}\;{\text{status }}\left( {\text{x}} \right) = \left\{ {\begin{array}{*{20}l} {{\text{if}}\;{\text{ x}} = 9\;} \hfill \\ {{\text{if }}\;1 \le {\text{x}} \le 8\;} \hfill \\ {{\text{if}}\;{\text{ x}} = 0\;{ }} \hfill \\ \end{array} } \right.\begin{array}{*{20}l} {{\text{Complete}}} \hfill \\ {{\text{Incomplete}}} \hfill \\ {{\text{None}}} \hfill \\ \end{array} $$

Lastly, any child that had received all the 9 basic childhood vaccines was classified as “1”, incompletely immunized as “2” and not immunized as “3”.

#### Explanatory variables

The basic explanatory variables are the individual and community-level characteristics. Individual-level variables included socio-demographic and economic characteristics such as the mother’s age in years, mother educational level, mother’s religious affiliation, mother’s occupational status, place of delivery, the total number of children ever born, wanted the last child, exposure to media, birth order, wealth quintile, sex of the child, number of antenatal and postnatal care attendance within 2 months. Exposure to media, in this study, was created from the combination of questions on the frequency of mothers’ access to information through newspapers/magazines, radio, or television. A set of variables including household assets, amenities and housing characteristics such as owner of television, refrigerator, type of flooring, source of drinking water, toilet facility, electricity, cooking gas and others were identified. The wealth index was constructed using principal component analysis (PCA). Each household in the dataset was assigned a wealth index score. These scores were then ranked and divided into quintiles and re-categorized into three wealth status; poor, average and rich. Community-level variables included the common characteristics of study subjects in an enumeration area such as place of residence, difficulty accessing health facilities, and state-level variables such as population density, number of healthcare facilities, and health facilities per 10,000 population.

### Data analysis procedure

The data was weighted due to the complex sampling strategy used for data collection and to compensate for the unequal probability of selection between clusters. Descriptive statistics were used to describe the data. High-resolution maps of coverage of each vaccine and dropout rates between doses (for vaccines that exist in doses) were used to assess geographical variation in access to and acceptance of routine immunization. These maps were also integrated with high-resolution population maps to produce estimates of the numbers of under-vaccinated children. SaTScan ArcGIS PRO 2.8 was used for spatial analysis and Stata 16.0 and R for non-spatial statistical analysis. SaTScan uses a circular window that moves systematically throughout the study area to identify statistically significant SaTScan clustering of areas with the same childhood basic vaccination coverage. Chi-square was used to examine factors that are associated with vaccination among children. At the level of multivariate analysis, binary logistic regression was used to identify factors influencing incomplete vaccination among children aged 12–23 months. In this study, the categories were dichotomized as 1 (if the child had completed the required vaccination) and 0 (if otherwise). A check of multicollinearity of the explanatory variables was carried out, and a condition number of 2.48 was obtained. This clearly indicated that the predicting factors have a low-level multicollinearity and are linearly independent. Thus, multilevel logistic regression models were generated and fitted to identify community and individual-level factors associated with childhood basic vaccination. The regression equation governing the use of multilevel modeling is given by Eq. (1):

1 $$ Y_{ij} = \beta_{0j} + \beta_{Ij} X_{Ij} + e_{ij} $$

where; $$\beta_{0j} = \gamma_{00} + \gamma_{01} Z_{j} + u_{0j}$$ and $$\beta_{ij} = \gamma_{10} + \gamma_{11} Z_{j} + u_{1j}$$.

$\gamma_{00}$ and $\gamma_{01}$ are the intercept and slope to predict $\beta_{0j}$ from $Z_{j}$, $u_{0j}$ is the residual error term in the equation for $\beta_{0j}$, $\gamma_{10}$ and $\gamma_{11}$ are the intercept and slope to predict $\beta_{ij}$ from $Z_{j}$ and $u_{ij}$ is the residual error term in the equation for $\beta_{ij}$. Three models were fitted: the first model with individual-level variables (model I), the second model with individual and community-level variables (Model II) and the third model (Model III) with all the individual, community, and state-level variables. These models are given in Eqs. (2–4)

2 $$ \log \left\{ { \frac{{p\left( {y_{k1} = 1} \right)}}{{1 - p\left( {y_{k1} = 1} \right)}}} \right\} = \beta_{2i} + \mathop \sum \limits_{{j_{n} = 1}}^{11} \beta_{2} x_{{j_{n} 2}} $$

3 $$ \log \left\{ { \frac{{p\left( {y_{k1} = 1} \right)}}{{1 - p\left( {y_{k1} = 1} \right)}}} \right\} = \beta_{3i} + \mathop \sum \limits_{{j_{n} = 1}}^{11} \beta_{3} x_{{j_{n} 2}} $$

4 $$ \log \left\{ { \frac{{p\left( {y = 1} \right)}}{{1 - p\left( {y = 1} \right)}}} \right\} = \beta_{0} + \beta_{1} x_{i1} + \beta_{2} x_{i2} + \cdots + \beta_{m} x_{im} $$

Model comparison was carried out using Bayesian Information Criteria (BIC) and Akaike’s Information Criterion (AIC). Finally, the model with the smallest value of the information criterion was selected as the best-fit model. Moreover, for measures of association (fixed effect), an adjusted odds ratio with 95% confidence intervals was used to determine statistical significance. For measures of variation (random effects), intra-class correlation coefficient (ICC), median odds ratio (MOR), and proportional change in variance (PCV) statistics were computed. ICC is a measure of within-cluster variation, the variation between individuals within the same cluster, and it was calculated using the formula: ${\text{ICC}} = \frac{{{\text{V}}_{{\text{A}}} }}{{{\text{V}}_{{\text{A}}} + \frac{{{\uppi }^{2} }}{3}}} = \frac{{{\text{V}}_{{\text{A}}} }}{{{\text{V}}_{{\text{A}}} + 3.29}}$, where ${\text{V}}_{{\text{A}}}$ is the estimated variance in each model. The total variation attributed to individual or/and community level factors at each model was measured by the proportional change in variance (PCV) and was calculated as $\frac{{{\text{V}}_{{\text{A}}} - {\text{ V}}_{{\text{B}}} }}{{{\text{V}}_{{\text{A}}} }}$, where ${\text{V}}_{{\text{A}}}$ = variance of the initial model, and ${\text{V}}_{{\text{B}}}$ = variance of the model with more terms^12^.

### The Bayesian Binary Regression model

The binary regression model^13^ is used to explain the probability of a binary response variable as function of some covariates. The model is specified by:

5 $$ y_{i} |\pi_{i} = Ber\left( {\pi_{i} } \right),\;\;\pi_{i} = \Pr \left( {y_{i} = 1} \right) = F\left( {{\varvec{X}}_{i}^{t} {\varvec{\beta}}} \right), $$

where $y_{i} = 1$ if the response of interest is observed for the $i{\text{th}}$ individual and zero otherwise, $\pi_{i}$ is the probability that the $i{\text{th}}$ individual presents the response under investigation, β is the *K* vector of unknown parameters, ${\varvec{X}}_{i}^{t} = \left( {x_{i1, } x_{i2, } \ldots , x_{iK, } } \right)$ the *K* vector of known covariates associated to the $i{\text{th}}$ individual and *F* any transformation assuming values in (0, 1). The function F, for example, could be any arbitrary cumulative distribution function. The logistic, normal, and extreme values are the most useful F specifications, which are:

6 $$ F\left( {X_{i}^{t} {\varvec{\beta}}} \right) = \left\{ {\begin{array}{*{20}l} {{{{\text{exp}}\left( {X_{i}^{t} {\varvec{\beta}}} \right)} \mathord{\left/ {\vphantom {{{\text{exp}}\left( {X_{i}^{t} {\varvec{\beta}}} \right)} {\left[ {1 + \exp \left( {X_{i}^{t} {\varvec{\beta}}} \right)} \right]}}} \right. \kern-0pt} {\left[ {1 + \exp \left( {X_{i}^{t} {\varvec{\beta}}} \right)} \right]}},} \hfill &\quad  {\left( {logistic} \right)} \hfill \\ { \varphi \left( {X_{i}^{t} {\varvec{\beta}}} \right),} \hfill &\quad  {\left( {probit} \right)} \hfill \\ {1 - \exp \left[ { - \exp \left( {X_{i}^{t} {\varvec{\beta}}} \right)} \right],} \hfill &\quad  {\left( {complementarylog - {\text{log}}} \right)} \hfill \\ \end{array} } \right\} $$

The link function defines the linear predictor as:

7 $$ \eta_{{\varvec{i}}} = \user2{ F}^{ - 1} \left( {\pi_{i} } \right) = \user2{ \beta }_{1} x_{i1} + \user2{ \beta }_{2} x_{i2 } + \user2{ } \cdots \user2{ } + \user2{ \beta }_{K} x_{iK} $$

For each of the three models stated above the linear predictor is respectively: $\log \left( {\pi_{i} /(1 - \pi_{i} } \right))$, ${\Phi }(\pi_{i} )$ and $\log ( - \log \left( {1 - \pi_{i} } \right))$. The model choice depends on the relation between the response variable and the covariates.

The likelihood function for data ${\varvec{y}} = \user2{ }\left( {y_{1} \user2{ },\user2{ }y_{2} \user2{ },\user2{ } \ldots ,\user2{ }y_{n} } \right)^{{\varvec{t}}}$ is

8 $$ p({\mathbf{y}}{|}\beta {)} = \mathop \prod \limits_{i = 1}^{n} [F\left( {X_{i}^{t} \beta } \right)]^{{y_{i} }} \left[ {1 - F\left( {X_{i}^{t} \beta } \right)} \right]^{{\left( {1 - y_{i} } \right)}} $$

It is important to establish a joint prior distribution over the parameter space in order to proceed with the Bayesian analysis. Because the relationship between the data and the parameters is so intricate, this is extremely difficult to accomplish. The easiest way to circumvent this difficulty is to propose an informative prior, but with small precision, avoiding any complaint about the specification of subjective beliefs^14^. In this study, informative independent normal priors, with extremely small precisions, was set to the parameters. Therefore,

9 $$ p(\beta {|}{\mathbf{y}}{)} \propto p\left( \beta \right)\mathop \prod \limits_{i = 1}^{n} [F\left( {X_{i}^{t} \beta } \right)]^{{y_{i} }} \left[ {1 - F\left( {X_{i}^{t} \beta } \right)} \right]^{{\left( {1 - y_{i} } \right)}} $$

Clearly,^9^ is a complex function of the parameters and numerical methods are needed in order to obtain the marginal posterior distribution for each of the model parameters. Simulation based methods have proliferated in the last ten years or so yielding two popular approaches known as *importance sampling*^15^ and *Gibbs sampling*^16,17^.

### Ethical consideration

The survey was conducted under relevant guidelines and regulations. Permission to use the data was sought and granted by the data originator. However, ethical approval to conduct the study was obtained from the National Ethical Review Committee (NREC), and informed consent was also obtained from the respondents before the conduct of the interview. The respondents were assured of the anonymity of the information they provided. The possible identifier that could be used to track each respondent to the information they provided was removed from the data before use.

## Results

**Figure 2:**
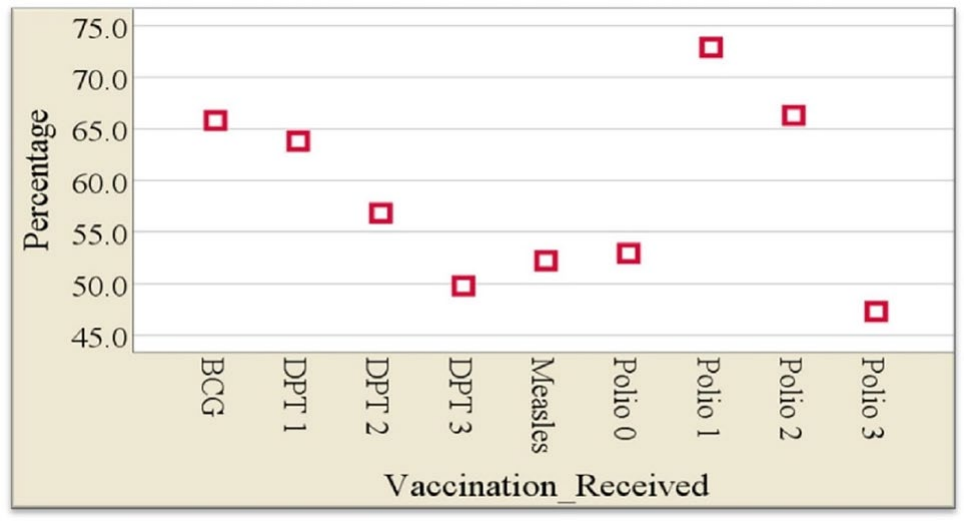
Percentage distribution of vaccines taken by children aged 12–23 months in Nigeria (2018).

The descriptive analysis of respondents by the vaccination status of children showed that a total of 5475 children were included in the study. 1074 (19.6%) children did not receive any of the recommended vaccines, 3053 (55.8%), representing more than half of the children were partially vaccinated and 1348 (24.6%) of the children were only fully vaccinated. Figure 2 presents the percentage distribution of children who took each of the vaccinations under consideration. About three-quarters (72.9%) of the children received the second dose of the Polio vaccine and more than half (52.7%) of the children did not receive Polio 3-the fourth dose of the Polio vaccine. Also, 50.2% of the children did not receive the third dose of Diphtheria Tetanus Pertussis (DPT3).

Except for the sex of the child, Table 1 revealed that the vaccination status of the children was significantly associated with all of the explanatory variables.

**Table 1:** Prevalence of vaccination status by socio-demographic factors in Nigeria.

	Vaccination status			p-value
	Complete	Incomplete	None	
	n (%)	n (%)	n (%)	
Individual level characteristics				
Sex of the child				
Male	697 (24.5)	1563 (55)	583 (20.51)	0.216
Female	651 (24.73)	1490 (56.61)	491 (18.66)	
Mothers’ Age (years)				
15 – 24	371 (18.56)	1153 (57.68)	475 (23.76)	< 0.001
25 – 34	651 (29.26)	1202 (54.02)	372 (16.72)	
35 – 49	326 (26.06)	698 (55.80)	227 (18.15)	
Level of education				
No formal education	249 (10.07)	1406 (56.88)	817 (33.05)	< 0.001
Primary	187 (23.40)	491 (61.45)	121 (15.14)	
Secondary/tertiary	912 (41.38)	1156 (52.45)	136 (6.17)	
Religion				
Christianity	766 (36.90)	1128 (54.34)	182 (8.77)	< 0.001
Islam	577 (17.17)	1894 (56.35)	890 (26.48)	
Others	5 (13.16)	31 (81.58)	2 (5.26)	
Occupation				
Unemployed/Unskilled manual	278 (29.80)	506 (54.23)	149 (15.97)	< 0.001
Employed	312 (38.76)	430 (53.42)	63 (7.83)	
Self employed	707 (19.64)	2,042 (56.72)	851 (23.64)	
Others	51 (37.23)	75 (54.74)	11 (8.03)	
Parity				
Low parity (parity 1 and 2)	554 (28.31)	1053 (53.81)	350 (17.88)	< 0.001
Multiparity (parity 3 and 4)	433 (26.66)	907 (55.85)	284 (17.49)	
Grand multiparity (parity 5 or more)	361 (19.06)	1093 (57.71)	440 (23.23)	
Wanted last child				
Wanted then	1169 (24.01)	2716 (55.78)	984 (20.21)	< 0.001
Wanted later	141 (32.19)	240 (54.79)	57 (13.01)	
Wanted no more	38 (22.62)	97 (57.74)	33 (19.64)	
Place of delivery				
Home or elsewhere	450 (13.87)	1843 (56.8)	952 (29.34)	< 0.001
Hospital/health center	898 (40.27)	1210 (54.26)	122 (5.47)	
Exposure to media				
Not exposed to media	737 (18.72)	2211 (56.17)	988 (25.1)	< 0.001
Exposed to media	611 (39.7)	842 (54.71)	86 (5.59)	
Wealth status				
Poor	298 (11.55)	1509 (58.51)	772 (29.93)	< 0.001
Average	267 (24.23)	656 (59.53)	179 (16.24)	
Rich	783 (43.65)	888 (49.5)	123 (6.86)	
Frequency of antenatal visits				
No visit	66 (4.85)	660 (48.53)	634 (46.62)	< 0.001
1–3 visits	173 (18.95)	581 (63.64)	159 (17.42)	
4–7 visits	677 (31.68)	1228 (57.46)	232 (10.86)	
8 or more visits	432 (40.56)	584 (54.84)	49 (4.6)	
Postnatal care attendance within 2 months				
No	905(20.94)	2404(55.62)	1013(23.44)	< 0.001
Yes	440 (38.26)	649 (56.43)	61 (5.3)	
Healthcare decision maker				
Respondent alone	149 (33.04)	260 (57.65)	42 (9.31)	< 0.001
Partner alone	596 (35.12)	909 (53.57)	192 (11.31)	
Respondent and partner	603 (18.12)	1884 (56.63)	840 (25.25)	
Region of residence				
North Central	264 (27.16)	572 (58.85)	136 (13.99)	< 0.001
North East	197 (16.29)	701 (57.98)	311 (25.72)	
North West	232 (14.34)	893 (55.19)	493 (30.47)	
South East	270 (47.45)	269 (47.28)	30 (5.27)	
South South	162 (31.15)	306 (58.85)	52 (10)	
South West	223 (37.99)	312 (53.15)	52 (8.86)	
Community level characteristics				
Place of residence				
Urban	739 (39.39)	926 (49.36)	211 (11.25)	< 0.001
Rural	609 (16.92)	2127 (59.1)	863 (23.98)	
Community poverty rate				
Low	147 (10.47)	941 (67.02)	316 (22.51)	< 0.001
Average	236 (16.19)	1021 (70.03)	201 (13.78)	
High	456 (17.45)	1675 (64.10)	482 (18.45)	
Community illiteracy rate				
Low	234 (15.86)	769 (52.14)	472 (32.00)	< 0.001
Average	378 (19.23)	1156 (58.83)	431 (21.93)	
High	409 (20.10)	1461 (71.79)	165 (8.11)	
Community unemployment				
Low	239 (14.14)	878 (51.95)	573 (33.91)	< 0.001
Average	203 (12.36)	997 (60.72)	442 (26.92)	
High	254 (11.85)	1602 (74.76)	287 (13.39)	
State level characteristics				
Rural proportion				
Low rural proportion (< 33.3%)	148 (14.08)	534 (50.81)	369 (35.11)	< 0.001
Average rural proportion (33.4–66.66%)	214 (16.16)	776 (58.61)	334 (25.23)	
High rural proportion (> 66.66%)	583 (18.81)	2072 (66.84)	445 (14.35)	
Health facility per 100,000				
< 15	345 (22.86)	867 (57.46)	297 (19.68)	< 0.001
15–25	349 (12.18)	1937 (67.61)	579 (20.21)	
> 25	203 (18.44)	655 (59.49)	243 (20.07)	

### Spatial prevalence of childhood vaccines uptake in Nigeria

**Figure 3:**
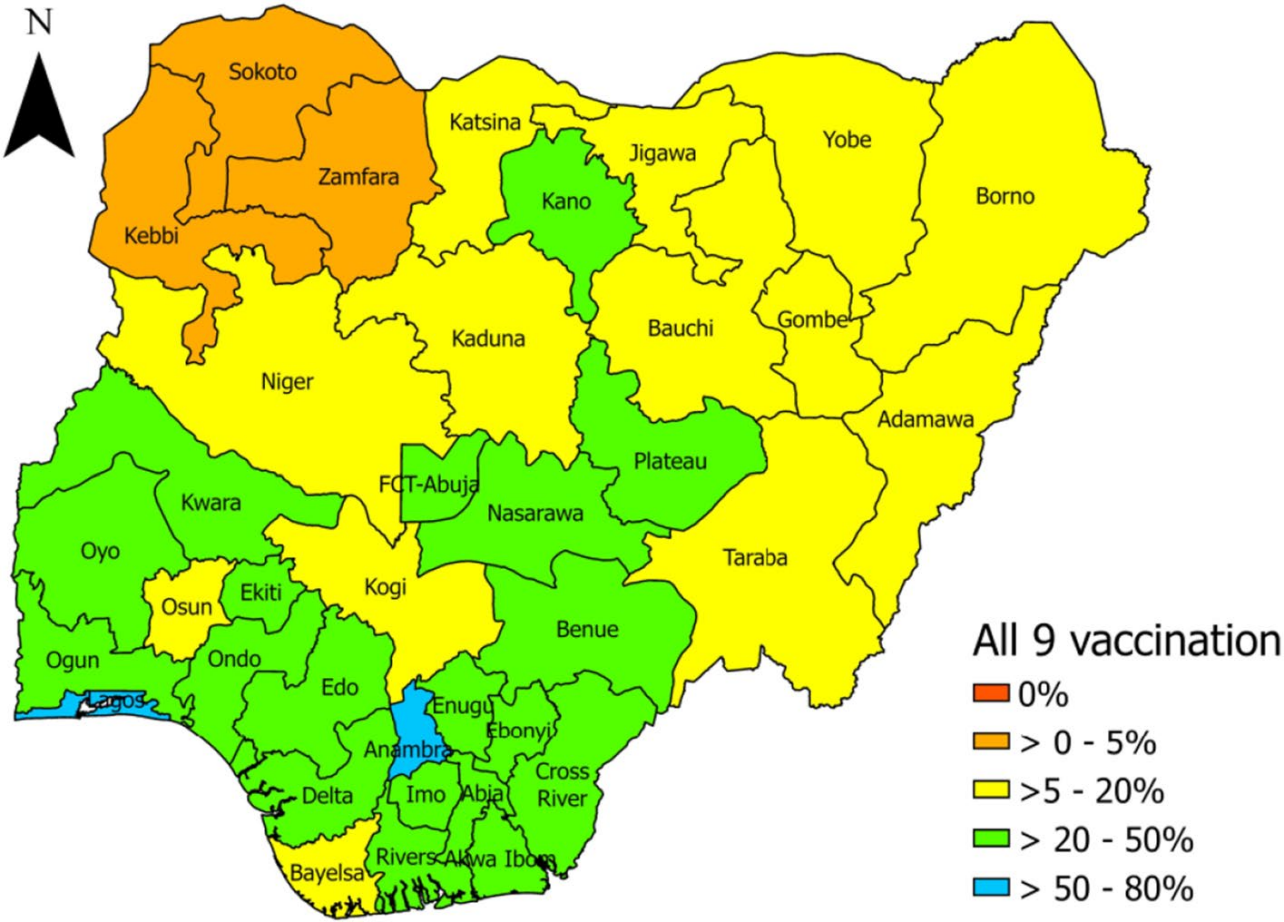
Spatial prevalence of complete vaccination in Nigeria (2018).

Figure 3 disaggregated the spatial prevalence of childhood vaccination. No state was found to have a 0% prevalence but Kebbi, Sokoto, and Zamfara states in the Northern region had the least (˂5%) prevalence of complete vaccination. The highest prevalence above 50 was observed in Lagos and Anambra states.

### Optimized hotspot analysis

**Figure 4:**
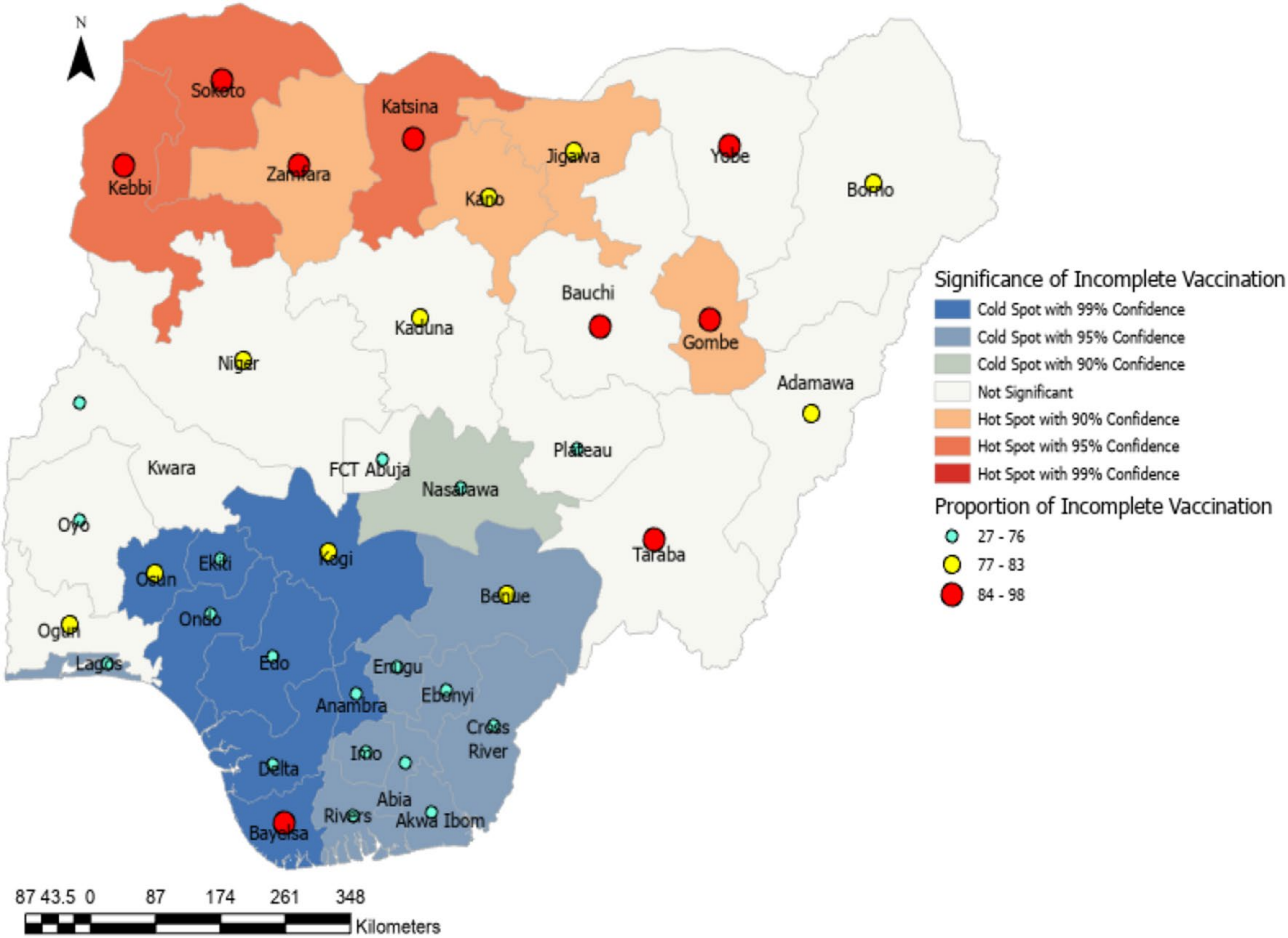
Optimized hotspot mapping of incomplete vaccination in Nigeria (2018).

The prevalence of incomplete vaccination among children aged 12–23 months in Nigeria is revealed in Fig. 4. Identifying clusters of various locations with a significantly high prevalence of incomplete vaccination is provided by this analysis. The significance of such locations is determined at a 95% confidence interval and areas with significantly high prevalence are referred to as hotspots.

### Random-intercept model of factors associated with childhood incomplete vaccination in Nigeria

The Multilevel Logistic model result is presented in this section. Model I (in Table 2) contains only the individual level variables, Model II contains an individual level and community level variables and Model III contains all levels’ variables. The AIC and BIC of the three models show that the difference between them is quite negligible as they are very close. Even though Model I is the lowest in terms of AIC and BIC but Model III will be used for the interpretation since it captures more information.

**Table 2:** Factors associated with childhood incomplete vaccination in Nigeria.

Characteristics	Model I	Model II	Model III
Fixed effects			
Individual level			
Sex of child			
Male	Ref (1)	Ref (1)	Ref (1)
Female	0.72 (0.41, 1.26)	0.71 (0.40, 1.25)	0.70 (0.40, 1.24)
Mother’s age			
15–24	Ref (1)	Ref (1)	Ref (1)
25–34	0.97 (0.56, 1.68)	0.95 (0.54, 1.65)	0.93 (0.53, 1.64)
35–49	1.67 (1.48, 1.93)	1.66 (1.48, 1.92)	1.66 (1.48, 1.92)
Maternal education			
No education	Ref (1)	Ref (1)	Ref (1)
Primary	1.16 (1.04, 1.52)	1.16 (1.04, 1.52)	1.15 (1.03, 1.50)
Secondary/tertiary	1.30 (1.13, 2.03)	1.52 (1.26, 2.42)	1.51 (1.15, 2.40)
Religion			
Christianity	Ref (1)	Ref (1)	Ref (1)
Islam	0.77 (0.55, 1.08)	0.73 (0.52, 1.03)	0.74 (0.53, 1.04)
Others	0.06 (0.01, 0.56)	0.06 (0.01, 0.54)	0.06 (0.01, 0.55)
Occupation			
Unemployed/unskilled	Ref (1)	Ref (1)	Ref (1)
Employed	1.16 (0.59, 2.28)	1.14 (0.58, 2.24)	1.15 (0.59, 2.26)
Self-employed	1.33 (0.34, 5.20)	1.35 (0.34, 5.30)	1.35 (0.34, 5.29)
Others	0.76 (0.22, 2.60)	0.74 (0.22, 2.53)	0.74 (0.22, 2.54)
Parity			
≤ 4	Ref (1)	Ref (1)	Ref (1)
5+	1.58 (0.88, 2.84)	1.67 (0.93, 3.00)	1.68 (0.93, 3.02)
Wanted last child			
Wanted then	Ref (1)	Ref (1)	Ref (1)
Wanted later	1.20 (0.88, 1.64)	1.21 (0.88, 1.65)	1.21 (0.88, 1.65)
Wanted no more	0.82 (0.49, 1.36)	0.81 (0.49, 1.35)	0.82 (0.49, 1.35)
Place of delivery			
Home or elsewhere	Ref (1)	Ref (1)	Ref (1)
Hospital/Health center	2.47 (1.79, 3.40)	2.47 (1.79, 3.41)	2.48 (1.80, 3.43)
Exposure to media			
Not exposed to media	Ref (1)	Ref (1)	Ref (1)
Exposed to media	1.40 (1.28, 1.58)	1.40 (1.27, 1.58)	1.40(1.27,10.58)
Wealth status			
Poor	Ref (1)	Ref (1)	Ref (1)
Average	1.27 (0.91, 1.77)	1.29 (0.91, 1.83)	1.28 (0.90, 1.82)
Rich	1.56 (1.11, 2.19)	1.56 (1.11, 2.20)	1.57 (1.12, 2.20)
Frequency of antenatal visits			
No visit	Ref (1)	Ref (1)	Ref (1)
1–3 visits	1.45 (1.10, 1.91)	1.48 (1.12, 1.96)	1.47 (1.12, 1.95)
4–7 visits	1.45 (1.21, 2.46)	1.47 (1.22, 2.46)	1.47 (1.22, 2.47)
8 or more visits	2.84 (1.96, 3.81)	2.84 (1.96, 3.82)	2.85 (1.95, 3.81)
Postnatal care attendance within 2 months after delivery			
No	Ref (1)	Ref (1)	Ref (1)
Yes	1.47 (0.68,2.66)	1.47 (0.67,2.67)	1.46 (0.68,2.67)
Don't know	0.65 (0.20, 3.99)	0.65 (0.21, 3.98)	0.64 (0.20, 3.99)
Healthcare decision maker			
Respondent alone	Ref (1)	Ref (1)	Ref (1)
Partner alone	0.89 (0.58, 1.77)	0.89 (0.77, 1.86)	0.87 (0.69, 1.94)
Respondent and partner	1.15 (0.82, 2.20)	1.27 (0.81, 2.31)	1.36 (0.79, 2.54)
Region			
North central	Ref (1)	Ref (1)	Ref (1)
North east	0.99 (0.36, 2.78)	0.89 (0.31, 2.50)	0.95 (0.34, 2.65)
North west	2.79 (1.0, 7.79)	2.50 (0.89, 7.08)	3.03 (0.99, 9.24)
South east	1.24 (0.36, 4.27)	1.24 (0.36, 4.26)	0.71 (0.18, 2.82)
South south	1.57 (0.53, 4.59)	1.60 (0.54, 4.73)	0.78 (0.20, 2.98)
South west	0.56 (0.19, 1.65)	0.54 (0.18, 1.59)	0.67 (0.22, 2.02)
Community level			
Place of residence			
Urban		Ref (1)	Ref (1)
Rural		1.14 (0.91, 1.42)	0.93 (0.69, 1.27)
Community poverty level			
Low		Ref (1)	Ref (1)
Average		1.04 (0.73, 1.47)	0.82 (0.42, 1.62)
High		1.63 (1.05, 2.53)	0.93 (0.63, 1.38)
Community illiteracy level			
Low		Ref (1)	Ref (1)
Average		0.95 (0.70, 1.29)	1.04 (0.74, 1.47)
High			1.64 (1.05, 2.55)
Community unemployment level			
Low		Ref (1)	Ref (1)
Average		0.97 (0.86, 2.17)	0.96 (0.85, 2.16)
High		0.96 (0.76, 2.29)	0.97 (0.75, 2.28)
State level			
Urban population			
Low rural prop (< 33.33%)			Ref (1)
Middle rural prop (33.34–66.66%)			0.97 (0.41, 2.34)
High rural prop (> 66.66%)			2.54 (0.79, 8.19)
Health facility			
< 15 per 100,000 pop			Ref (1)
15–25 per 100,000			1.24 (0.88, 1.96)
> 25 per 100,000			1.62 (0.91, 2.47)
State population			
< 3 million			Ref (1)
3–6 million			0.97 (0.69, 2.02)
> 6 million			0.86 (0.45, 1.89)
Random effects			
Variance			
Community	0.89 (0.59, 1.34)	0.87 (0.57, 1.32)	0.87 (0.57, 1.33)
States	0.81 (0.46, 1.42)	0.81 (0.46, 1.41)	0.76 (0.43, 1.34)
ICC			
Community	0.45 (0.04, 0.26)	0.45(0.04, 0.26)	0.43 (0.04, 0.26)
States	0.20(0.04, 0.10)	0.20(0.04, 0.10)	0.19 (0.04, 0.09)
Diagnostics			
AIC	*− 4316.225*	*− 4319.225*	*− 4322.715*
BIC	*− 4725.668*	*− 4736.312*	*− 4782.101*

Significant values are in italics.

Mother’s age and education, religion, place of delivery, media exposure, household wealth status, frequency of antenatal visits, postnatal care attendance within 2 months of delivery, community poverty level, state health facility, and population were all found to be significantly associated with childhood incomplete vaccination. Children whose mothers’ ages fall between 35 and 39 years were found to have higher odds (OR = 1.66, 95% CI: 1.48, 1.92) of complete vaccination compared to children whose mothers fall in the age group 15–24 years. Women with primary and secondary/tertiary education were found to have higher odds [(OR = 1.15, 95% CI: 1.03, 1.50) and (OR = 1.51, 95% CI: 1.15, 2.40) respectively] of complete vaccination for their children compared to women who had no formal education. Children whose mothers practice none of Christianity or Islam religion were found to have lower odds of complete vaccination compared to those who practice Christianity (OR = 0.06, 95% CI: 0.01, 0.55).

Likewise, children who were delivered in the hospital/health center were found to have higher odds of (OR = 2.48, 95% CI: 1.80, 3.43) of complete vaccination relative to children who were delivered at home or elsewhere. Women who were exposed to media were also found to have higher odds of 40% (OR = 1.40, 95% CI: 1.27, 10.58) of complete vaccination for their children compared to women who were not exposed to media. Antenatal and Postnatal care attendance was also found to be significant. Children whose mothers went for 1–3, 4–7, and above 8 antenatal visits were found to have higher odds [(OR = 1.47, 95% CI: 1.12, 1.95), (OR = 1.47, 95% CI: 1.22, 2.47) and (OR = 2.85, 95% CI: 1.95, 3.81) respectively] of complete vaccination compared to children whose mothers did not go for any antenatal visit. Those who went for postnatal care attendance within two months of delivery had higher odds of 46% (OR = 1.46, 95% CI: 0.68, 2.67) of complete vaccination for their children relative to children whose mothers did not go for any postnatal care attendance. According to Model III, the community and state random levels contribute 43% and 19% variation respectively to the effects of factors associated with childhood incomplete vaccination in Nigeria.

### Integrated nested Laplace approximation diagnostics on factors associated with vaccination status among children aged 12–23 months in Nigeria

Integrated nested Laplace approximation (INLA) offers an alternative to the computationally intensive Markov chain Monte Carlo (MCMC) methods^18,19^. INLA focuses on obtaining an approximation of the posterior marginal distributions of the model's parameters. To check whether the quality of the sample generated with the INLA algorithm was sufficient to provide an accurate approximation of the target distribution, the integrated nested Laplace approximation (INLA) diagnostics were employed.

Two diagnostics tools were used; the dispersion check and the distribution check. The classic dispersion measure is to sum the squared Pearson residuals and divide them by the number of observations minus the degrees of freedom. The dispersion check (D = 0.87) showed that there were no overdispersion and underdispersion, and no apparent anomalies in the chain, and the chain seemed to extensively explore the sample space many times. The distribution check takes the marginal distribution of the fitted values into account and returns the data frame with the empirical cumulative distribution of the raw data (ecdf) and the envelope of empirical cumulative distribution from data simulated from the model. The ratio of the ecdf of the raw data to the ecdf of the simulated data is near 1 (0.847). The model makes sense since the observed ecdf is within the envelope of the simulated ecdf.

Table 3 below presents the adjusted odds of factors associated with incomplete vaccination among children aged 12–23 months in Nigeria. Children of mothers aged 25–34 and 35–49 had lower odds of 33% and 41% (aOR = 0.67, 95% CI: 0.54, 0.82 and aOR = 0.59, 95% CI: 0.46, 0.77) to be incompletely vaccinated compared to those of mothers in the age group 15–24. There is also a lower odds of 28% (aOR = 0.72, 95%CI: 0.54, 0.95) of being incompletely vaccinated by children whose mothers had primary education compared to those children whose mothers had no formal education.

**Table 3:** Bayesian model of factors associated with childhood incomplete vaccination in Nigeria.

Background characteristics	Model I	Model II	Model III
Characteristics	aOR (95% C.I)	aOR (95% C.I)	aOR (95% C.I)
Individual level			
Sex of child			
Male	1.00	1.00	1.00
Female	0.97 (0.83, 1.12)	0.96 (0.83, 1.11)	0.96 (0.83, 1.11)
Mother’s age (years)			
15–24	1.00	1.00	1.00
25–34	0.66 (0.54, 0.81)^a^	0.67 (0.54, 0.82)^a^	0.67 (0.54, 0.82)^a^
35–49	0.59 (0.45, 0.76)^a^	0.59 (0.46, 0.77)^a^	0.59 (0.46, 0.77)^a^
Maternal education			
No education	1.00	1.00	1.00
Primary	0.64 (0.50, 0.83)^a^	0.72 (0.54, 0.95)^a^	0.72 (0.54, 0.95)^a^
Secondary/tertiary	0.45 (0.35, 0.58)^a^	0.51 (0.38, 0.67)^a^	0.50 (0.38, 0.67)^a^
Religion			
Christianity	1.00	1.00	1.00
Islam	1.09 (0.86, 1.39)	1.09 (0.85, 1.39)	1.08 (0.85, 1.38)
Others	2.00 (0.68, 6.69)	1.94 (0.66, 6.50)	2.00 (0.67, 6.69)
Occupation			
Unemployed/unskilled	1.00	1.00	1.00
Employed	0.98 (0.77, 1.25)	0.97 (0.76, 1.24)	0.98 (0.77, 1.25)
Self-employed	1.05 (0.86, 1.28)	1.03 (0.84, 1.26)	1.04 (0.85, 1.28)
Others	0.91 (0.59, 1.41)	0.92 (0.60, 1.43)	0.92 (0.59, 1.43)
Parity			
≤ 4	1.00	1.00	1.00
5 +	1.13 (0.11, 6.23)	1.29 (0.21, 4.20)	1.05 (0.10, 3.90)
Wanted last child			
Wanted then	1.00	1.00	1.00
Wanted later	0.94 (0.73, 1.21)	0.94 (0.73, 1.22)	0.93 (0.73, 1.21)
Wanted no more	1.59 (1.03, 2.48)^a^	1.60 (1.04, 2.51)^a^	1.59 (1.03, 2.49)^a^
Place of delivery			
Home or elsewhere	1.00	1.00	1.00
Hospital/Health center	0.63 (0.53, 0.76)^a^	0.64 (0.53, 0.77)^a^	0.64 (0.53, 0.76)^a^
Exposure to media			
Not exposed to media	1.00	1.00	1.00
Exposed to media	0.54 (0.44, 0.67)^a^	0.65 (0.57, 0.78)^a^	0.63 (0.54, 0.79)^a^
Wealth status			
Poor	1.00	1.00	1.00
Middle	0.60 (0.44, 0.82)^a^	0.82 (0.55, 1.21)	0.82 (0.55, 1.21)
Rich	0.41 (0.29, 0.59)^a^	0.58 (0.38, 0.89)^a^	0.58 (0.38, 0.89)^a^
Antenatal visits			
None	1.00	1.00	1.00
1–3	0.36 (0.26, 0.50)^a^	0.37 (0.27, 0.51)^a^	0.37 (0.27, 0.52)^a^
4–7	0.26 (0.19, 0.35)^a^	0.26 (0.19, 0.36)^a^	0.27 (0.20, 0.36)^a^
8 +	0.31 (0.22, 0.43)^a^	0.31 (0.22, 0.44)^a^	0.32 (0.23, 0.44)^a^
Postnatal care attendance within 2 months			
No	1.00	1.00	1.00
Yes	0.65 (0.54, 0.78)^a^	0.65 (0.54, 0.78)^a^	0.65 (0.54, 0.78)^a^
Healthcare decision maker			
Respondent	1.00	1.00	1.00
Respondent and Partner	0.89 (0.68, 1.17)	0.89 (0.67, 1.16)	0.87 (0.66, 1.14)
Partner alone	1.07 (0.82, 1.40)	1.07 (0.81, 1.39)	1.04 (0.79, 1.35)
Region			
North central	1.00	1.00	1.00
North east	1.08 (0.58, 2.01)	1.02 (0.55, 1.90)	0.95 (0.50, 1.80)
North west	1.18 (0.64, 2.19)	1.10 (0.60, 2.04)	1.42 (0.65, 3.13)
South east	0.95 (0.49, 1.84)	1.01 (0.52, 1.96)	1.09 (0.55, 2.15)
South south	1.32 (0.69, 2.52)	1.37 (0.72, 2.61)	1.22 (0.58, 2.60)
South west	1.55 (0.82, 2.93)	1.61 (0.86, 3.05)	2.12 (1.07, 4.24)^a^
Community level			
Place of residence			
Urban		1.00	1.00
Rural		1.12 (0.91, 1.39)	1.10 (0.89, 1.36)
Community poverty level			
Low		1.00	1.00
Average		1.19 (0.87, 1.62)	1.19 (0.87, 1.63)
High		1.36 (0.96, 1.93)	1.34 (0.94, 1.91)
Community illiteracy level			
Low		1.00	1.00
Average		1.08 (0.84, 1.40)	1.07 (0.83, 1.38)
High		1.41 (0.93, 2.13)	1.40 (0.92, 2.11)
Community unemployment level			
Low		1.00	1.00
Average		0.94 (0.76, 1.17)	0.93 (0.75, 1.16)
High		0.95 (0.75, 1.19)	0.94 (0.74, 1.18)
State level			
Urban population			
Low rural prop (< 33.33%)			1.00
Middle rural prop (33.34–66.66%)			1.50 (0.77, 2.88)
High rural prop (> 66.66%)			1.38 (0.72, 2.66)
Health facility			
< 15 per 100,000			1.00
15–25 per 100,000			1.12 (0.68, 1.83)
> 25 per 100,000			1.02 (0.51, 2.04)
State population			
< 3 million			1.00
3–6 million			0.92 (0.58, 1.45)
> 6 million			0.36 (0.15, 0.90)^a^

^a^Adjusted odds ratio significant at 95% confidence interval.

Similarly, the odds of childhood incomplete vaccination is 36% lower (aOR = 0.64, 95%CI: 0.53, 0.76) among children who were delivered in a hospital or other health center compared with children who were delivered at home or elsewhere. Children whose mothers were exposed to media (television, radio, and newspaper) had a lower odds of 37% (aOR = 0.63, 95%CI: 0.54, 0.79) to be incompletely vaccinated compared to the children whose mothers were not exposed to media. Household wealth status where a child belongs is a significant factor associated with incomplete vaccination. Children who belong to a household whose wealth status category is rich had lower odds of 42% (aOR = 0.58, 95%CI: 0.38, 0.89) of being incompletely vaccinated compared to children with poor household wealth status.

Additionally, there are lower odds of 63% (aOR = 0.37, 95%CI: 0.27, 0.52), 73% (aOR = 0.27, 95%CI: 0.20, 0.36), and 68% (aOR = 0.32, 95%CI: 0.23, 0.44) among children whose mothers went for 1–3, 4–7 and more than 8 antenatal visits respectively for incomplete vaccine uptake compared to children whose mothers did not go for any antenatal visit. Similarly, children whose mothers attended postnatal care within 2 months of delivery had lower odds of 35% (aOR = 0.65, 95%CI: 0.54, 0.78) to be incompletely vaccinated compared to those children whose mothers did not attend any postnatal care within 2 months of delivery. Likewise, children whose mothers reside where the state population is greater than 6 million had lower odds of 64% (aOR = 0.36, 95%CI: 0.15, 0.90) of incomplete vaccination compared to children whose mothers reside where the state population is less than 3 million.

### Posterior prediction of vaccination status among children aged 12–23 months in Nigeria

**Figure 5:**
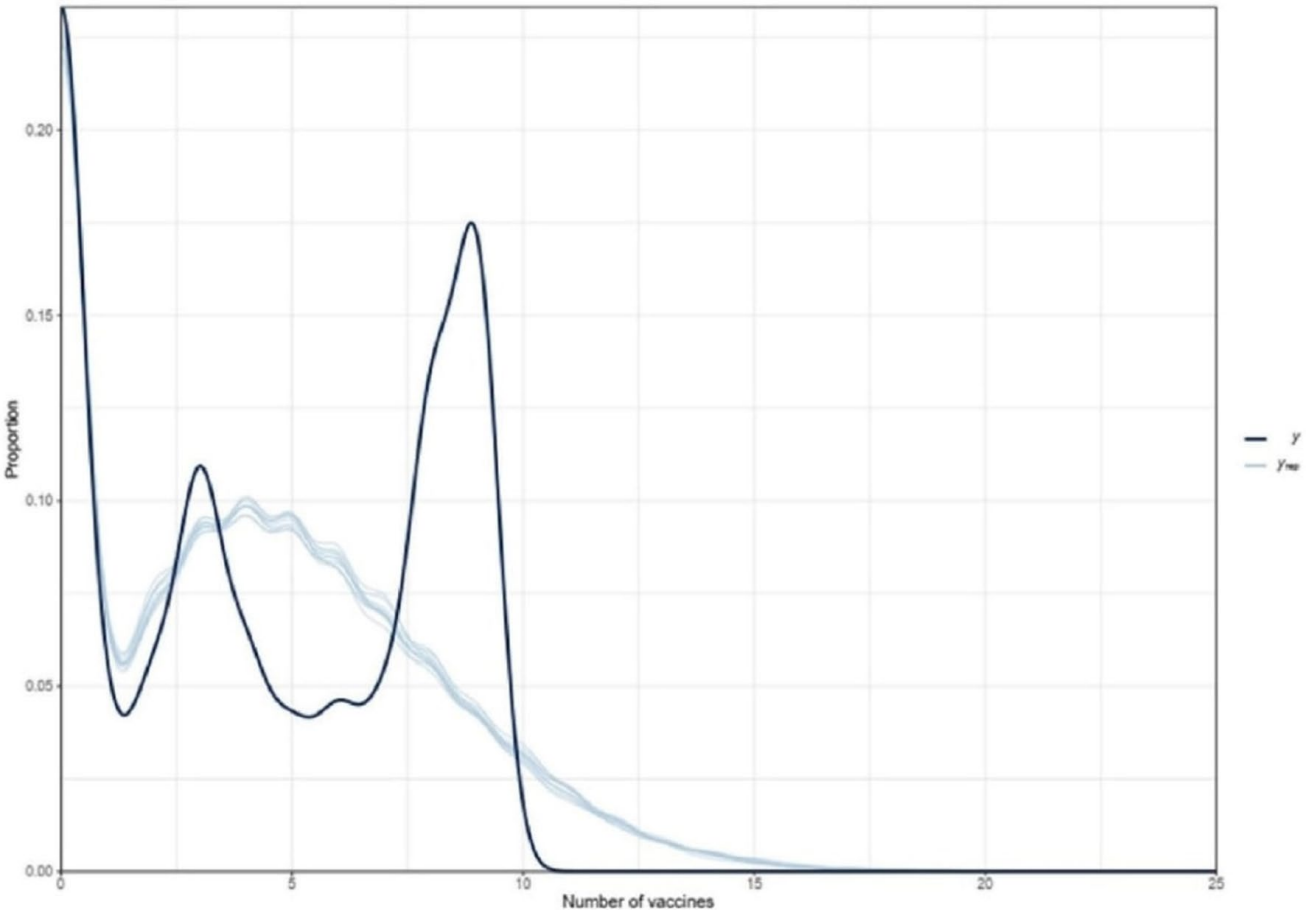
Plot of posterior predicts of the number of vaccinations among children aged 12–23 months in Nigeria (2018).

The graph below (Fig. 5) is the posterior predictive check, which helps in inspecting the postulated or predicted values of vaccine uptake among children aged 12–23 months in Nigeria, against the observed values from the data. After using the observed data and predictors, we can update our belief about the unknown parameter (keeping all variables constant). The result predicts a continuous high number of children with no vaccination (over one-fourth of the population), then a sharp drop in the number of people that received one vaccination. Hereafter, a slight increase in the number of children taking 2 or 3 vaccinations (about 10%), accompanied by a steep decline in later vaccinations. This is contrary to the increase observed in the data after the 5th dose of vaccination.

## Discussion

The prevalence of incomplete vaccination in Nigeria was high but strikingly higher in the Northern regions than in the regions in the South. This is due to relatively high level of non-formal education and low level of exposure to media of mothers in the north as compared to those in the southern parts of the country. This reveals that the mothers’ educational level as a predisposing factor according to the Andersen’s healthcare utilization model, influences children vaccines uptake behaviour. The findings of Adedokun et al.^20^ and Afolabi et al.^11^ corroborate with this assertion. The study revealed that one in every four children aged 12–23 months was not fully vaccinated in Nigeria. This finding is in agreement with the outcomes of the studies conducted by Sato and Eze et al., where similar level was observed^6,7,21^. The high level of incomplete vaccination observed in Nigeria could be possibly due to the inaccessibility of vaccination officers to some areas and localities where security issues were of serious challenge. With Andersen’s healthcare utilization model, an enabling factor like healthcare providers is also found to impact childhood immunization rate in Nigeria. The perception of people about vaccination can be another possible reason for low vaccination coverage in Nigeria. When compared to children delivered at home or elsewhere, children delivered at health facilities receive all immunizations at birth; this is also reflected in an increased incidence of vaccination among women who attended antenatal care, and most importantly, postnatal care attendance within two months. Attendance in antenatal and postnatal clinics may be connected to a higher level of trust and confidence in the healthcare system. This finding is also in agreement with the study by Asuman et al.^22^ who found that religion and household wealth are significant drivers and predictors of immunization and other health-seeking behavior.

The Bayesian output revealed that the states in the Northern part of Nigeria are mainly hot spots for incomplete vaccination. Although the distribution of incomplete vaccination spread across the country, it is worthwhile to note that childhood vaccination coverage is fair in the South-west. This study found that individual characteristics, community factors, and state factors all had a significant role in determining the variation in children's incomplete immunization status in Nigeria. Children of young mothers (15–24 years) are more likely to be under-immunized as compared to children of older women. This is in line with earlier studies^6,21,23–25^. This indicates that when a mother’s age increases, the chance of her child receiving immunizations increases. The young mother’s inexperience with child care may be to blame for this issue. Older women have faced the strains of caring for ailing children, with the ensuing effects on time and household finances. These mothers would support any effort to stop childhood illnesses.

The study establishes the significance of education in predicting childhood immunization. The likelihood of having a child who is not completely vaccinated falls for a mother who just has elementary education or none at all compared to a child whose mother has secondary or higher education. It has been demonstrated that education significantly affects a mother’s health-seeking behavior, including child vaccination^1,26,27^. According to comparable research, household poverty has an impact on children's vaccination rates^1,6,21^. A family’s children are more likely to be under-immunized and unprotected against illnesses that can be prevented by vaccination the poorer the household grows.

The results of this study also demonstrate that pregnant women who use healthcare facilities help to raise the vaccination rates of their children. Children whose mothers received antenatal care had a decreased chance of not receiving the recommended vaccinations than children whose mothers did not get any prenatal care. This is also the result of earlier studies^27,28^. Attending prenatal care puts women in a better position to learn enough about routine childhood vaccination.

A mother also goes through processes during antenatal care that educates her to have a positive attitude toward using health care, not just for herself but also for her children. According to earlier studies, health facility delivery has a significant impact on baby immunization^1,6,21,29^. Incomplete vaccination is less common in children whose mothers gave birth in a hospital. Women who give birth in a health facility can learn about their child’s immunization regimen and get their infant immunized at birth.

The ease of access mothers had to medical facilities was associated with childhood immunization. As previously reported, children of mothers who had trouble attending medical facilities are more likely to receive only partial vaccinations^8,21,23,30^. Children whose mothers live in socioeconomically poor regions and states are more likely to be partially immunized. The findings of earlier studies^6,21,23^ indicating the socioeconomic makeup of communities affected people's health-seeking behavior support this.

### Limitations of the study

The study relied on secondary data. It is vulnerable to bottlenecks, omission mistakes, and manipulations because it is a secondary and retrospective cross-sectional survey (recall bias and no means of verification of all information supplied). Mothers’ recall bias, which is often induced by the lack of vaccination cards, could lead to underestimating the study, albeit the NDHS methodology would mitigate the implications of these estimations. Vaccination coverage rates, on the other hand, are usually a measure of completing the specified vaccination schedule rather than of protection (the indicator does not reflect if the vaccines were given at the recommended ages and recommended intervals). Thus, the temporal distribution as well as the hierarchical nature of the number of vaccines taken and associated factors were not accounted for in the study.

## Conclusions

This study considered the INLA random effects model to examine the factors associated with incomplete vaccination among children aged 12–23 months in Nigeria using the 2018 NDHS dataset. The performance of the model depended on the number of recommended vaccine uptake received by the children measured at different levels. A considerable number of children are not fully vaccinated against childhood infections in Nigeria. Individual and contextual factors significantly contributed to incomplete vaccination among children in the country. Therefore, the government and other stakeholders should pay particular attention to antenatal and postnatal attendance counseling programs for child-bearing mothers in all urban and rural parts of the country to overcome the problem of children’s incomplete vaccination and/or vaccination dropout. Vaccines are a successful method for reducing child morbidity and mortality in nations all over the world, yet vaccination rates in Nigeria are extremely low. To boost the odds of uptake after birth, programs focused on encouraging and educating pregnant women, as well as young women, should be introduced. To meet the aforementioned recommendations, the government should enlist the help of non-governmental organizations and other relevant groups in promoting and maintaining vaccination uptake among children, particularly in low-uptake areas.

## Data Availability

The dataset generated and analysed during the current study is available in the Measure DHS repository, https://www.dhsprogram.com.
